# Perspectives on Complexity, Chaos and Thermodynamics in Environmental Pathology

**DOI:** 10.3390/ijerph18115766

**Published:** 2021-05-27

**Authors:** Maurizio Manera

**Affiliations:** Faculty of Biosciences, Food and Environmental Technologies, University of Teramo, St. R. Balzarini 1, 64100 Teramo, Italy; mmanera@unite.it; Tel.: +39-0861-266980

**Keywords:** fractal, entropy, life, living organisms, system biology, biological interfaces

## Abstract

Though complexity science and chaos theory have become a common scientific divulgation theme, medical disciplines, and pathology in particular, still rely on a deterministic, reductionistic approach and still hesitate to fully appreciate the intrinsic complexity of living beings. Herein, complexity, chaos and thermodynamics are introduced with specific regard to biomedical sciences, then their interconnections and implications in environmental pathology are discussed, with particular regard to a morphopathological, image analysis-based approach to biological interfaces. Biomedical disciplines traditionally approach living organisms by dissecting them ideally down to the molecular level in order to gain information about possible molecule to molecule interactions, to derive their macroscopic behaviour. Given the complex and chaotic behaviour of living systems, this approach is extremely limited in terms of obtainable information and may lead to misinterpretation. Environmental pathology, as a multidisciplinary discipline, should grant privilege to an integrated, possibly systemic approach, prone to manage the complex and chaotic aspects characterizing living organisms. Ultimately, environmental pathology should be interested in improving the well-being of individuals and the population, and ideally the health of the entire ecosystem/biosphere and should not focus merely on single diseases, diseased organs/tissues, cells and/or molecules.

## 1. Introduction

Querying a scientific, biomedical search engine such as PubMed^®®^ for the keywords (as text word in title, abstract, Medical Subject Headings terms and subheadings) from the present title may return different results, depending on how the search string is composed. A single word search returned, respectively, 9217 results for “chaos”, 158,255 results for “complexity”, 100,872 results for “thermodynamics”, but only 44 results for “environmental pathology” (last accessed on 9 April 2021). Unfortunately, the combined search of all four keywords returned no result and the same held true for combining the latter with each single previous keyword. Omitting the term “environmental” from the cumulative search returned only one result [1]. These findings prove a general interest in biomedical sciences for chaos theory, complexity and thermodynamics, though such topics have not so far fascinated pathologists in general and environmental pathologists in particular. Certainly, this is not due to a lack of advantages in the comprehension of pathophysiology processes, but rather to a somewhat traditional and conservative approach of pathologists to their topics [2,3]. In fact, complexity science and chaos theory have gone far beyond scientific, academic interest, becoming a common scientific divulgation theme. Nevertheless, medical disciplines, and pathology in particular, still rely on a deterministic, reductionistic approach and still hesitate to fully appreciate the intrinsic complexity of living beings, aside from some noticeable exceptions [4,5,6,7,8,9].

Herein, complexity, chaos and thermodynamics are introduced with specific regard to living organisms and biomedical sciences; then, their interconnections and implications in environmental pathology are discussed, with particular regard to a morpho-pathological, image analysis-based approach.

## 2. Complexity, Chaos and Thermodynamics in Living Systems

### 2.1. Complexity

To date there is no univocal, inclusive, exhaustive definition of complexity. Nevertheless, a complex system may be considered as a system whose behaviour is not simply derivable from the behaviour of its single parts (the whole is greater than the sum of its parts), due to the phenomenon of “emergence” as the collective, consequent behaviour of these parts interacting with each other. Accordingly, the amount of information needed to detail the behaviour of such system can be referred to a measure of its complexity [10,11,12,13,14]. Referring to the theory of communication, the more the entropy of a system increases, the more the related informative content decreases [15]. The latter concept will be further expanded in the paragraph about thermodynamics. The computational, informative, physical measure of complexity, among others, are not satisfactory in describing living systems, where no definitive definition/measure is available [10,11,12,13,14]. Nevertheless, fractal dimension, as a possible measure of morphological complexity, has been used in pathology [8,16].

Apart from emergence, as a property of all complex systems, living systems also display “self-organizing” behaviour, referring to the capacity of controlling their own behaviour without the need of internal or external control, adaptive properties (complex adaptive system) and macroscopic complex changing behaviour (dynamic systems) [14]. It should be stressed that self-organization concept was primarily conceptualized by researchers in mathematics and physics, and should be referred to as possible intrinsic properties of physics and chemistry, rather than a complementary mechanism of evolution alongside natural selection, though some authors hope for a possible synthesis [17]. To some extent, this latent physicism was appreciable in the most famous work of D’Arcy Wentworth Thompson, On Growth and Form [18]. Another peculiarity of living organisms, as complex adaptive systems, is that they thrive at the edge of order and disorder [14,19]. In fact, starting from perfectly ordered and simple systems, a peak of complexity is reached where some underlying rule is assumed, which then decreases the more the disorder increases, assuming no underlying rule exists (stochastic systems) [10]. This borderline behaviour is extremized in chaotic systems. Moreover, such somewhat biphasic order to complexity diagram leads to the idea that disease has to be primarily regarded as a reduction/loss of inherent complexity, rather than a mere reduction/loss of order. As a consequence, fractal analysis has been proposed as a method to evaluate the complexity reduction associated to pathological changes in tissues [8,16,20,21].

Starting from Charles Darwin, biologists are seeking for general, universal properties among living organisms and, in particular, molecular biology tried to explain such properties in terms of molecules, with particular emphasis to DNA, as the universal language of transmission of the biological information through generation and as a possible source of variation during evolution. This is according to the following logical approach: organisms are approached at the microscopic, molecular level, with particular regard to the interactions among molecules, to the function associated with such interactions and to the possible DNA-related variations. As a consequence, the macroscopic behaviour of an organisms is deduced from the possible ways the constituting molecules combine with each other. Though the molecular approach has proven useful for medical implications, unfortunately it is at the present ineffective to explain real-life complexity, being extremely reductionistic and potentially effective only in rare, if any, one-to-one molecule interactive systems [13]. To put it simply, the excessive attention paid to fine molecular details may lead someone to “not see the wood for the trees”. More recently, in the post-genomic era, network analysis has been applied at different hierarchical levels in systems biology to study organisms as whole integrated systems, and to study how protein–protein interaction network (“interactome”) is related to functions [22,23,24].

### 2.2. Chaos

The scientific method relies on the logical succession of the following main steps: phenomena observation, proposing explanations, testing explanations. Proposing explanations implies the translation of the observed phenomena into a model, in particular, a mathematical one [5,25]. When applicable natural phenomena are approximated to linear models, where proportionality exists between causes and effects, and where each component can be analyzed separately and its effects summed algebraically to obtain the cumulative effects (the whole is equal to the sum of its parts). If linear models are not suitable to explain phenomena, more complex nonlinear models may be required, where components cannot be analyzed separately and algebraically summed to obtain a systemic effect (the whole is greater than the sum of its parts) [26].

In spite of this, the fundamental reductionist assumption is based on the knowledge of the initial conditions, given which the future behavior of the system can be described in a deterministic manner. Nevertheless, even if we were able to track each particle in the universe at the subatomic level, according to the concept of universal determinism of Laplace [27], we would be unable to predict any physical phenomena, biologically included [5]. According to Heisenberg, there is a fundamental precision limit in the predictability of the values for definite pairs of complementary variables (position and momentum of a particle) starting from initial condition. In fact, Heisenberg’s uncertainty principle “states a fundamental property of quantum systems and is not a statement about the observational success of current technology*”* [28]. Aside from quantum physics, generally speaking, the experimental act of observing reality implies an inevitable impact on the latter, unpredictably affecting the resulting measure (observer effect). The observer effect is instrument-related, which is notably different from the uncertainty principle [29,30].

In system modeling, many differential equations may lead to approximate solutions. Moreover, both analytic and numerical methods rely on irrational number, implying the practical need of rounding to a significant figure. In systems characterized by sensitivity to initial conditions, as stressed for the first time by Poincaré, such approximation may result in drastic variation in the predicted behavior [5,31,32,33]. As observed and stressed by Lorenz in weather forecast modeling (a typical example of complex dynamic system modelling), the iterative processes of nonlinear systems exponentially amplify such small initial differences that, contrary to linear systems, where differences in the initial conditions proportionally affect the predicted behavior, disproportionate effects may result [5,34]. This means that chaotic systems are inheritably unpredictable, though it does not imply they are not cognoscible or behave randomly. The apparently contradictory term “deterministic chaos” has been introduced to denote chaotic behavior expressed by nonlinear systems, where the time evolution of their state can be uniquely determined from their initial conditions, according to proper dynamical laws [33].

Among others, chaotic systems can be characterized in terms of strange attractors, bounded regions of the phase space (a multidimensional space, whose axes correspond to each coordinate necessary to describe the state of a physical system) where the trajectories generated by equations converge, forming loops without intersecting one another. Interestingly, strange attractors have fractal dimensions [5,33]. According to Prigogine, rather than referring to trajectories and points in the phase space, one should refer to a region defined in terms of probability distribution [35]. The conceptual and substantial difference between deterministic chaos, as previously defined, and stochastic chaos, definable in terms of state transitions, rather than in terms of dynamics, should be stressed [36,37].

Living organism displays chaotic behavior at different levels of organization, and both physiology and pathophysiology, e.g., cardiovascular, nervous and respiratory systems, can be approached by methods of nonlinear (chaotic) dynamic systems [26,33,38,39,40,41,42,43,44,45,46]. Accordingly, pathological state can be ascribed to the alteration of the attractor’s basin of attraction, and also in the transition from a (chaotic) strange attractor to (periodic) limit cycle or to a fixed point attractor [47,48,49]. The end of a biological function, and ultimately death, may be visualized as the rest point of a fixed-point attractor.

Network biology modeling has been proposed to identify the transition from normal to disease state in complex diseases, corresponding to the bifurcation point in dynamical systems theory. In particular, three states are recognized during progression: normal, pre-disease and disease state. Both normal and disease states are steady states characterized by high resilience and robustness. On the contrary, the pre-disease state shows low resilience and robustness. Drastic drop of state-transition-based local network entropy, as a dynamical network biomarker criterion, ensures the identification of pre-disease state, serving as a general early-warning indicator of imminent transitions, where traditional biomarkers fail [50].

### 2.3. Thermodynamics

Thermodynamics deals with the physical properties of systems of matter and energy. Though originally macroscopic, thermodynamics turned microscopic with the statistical mechanics approach of Boltzmann, where the emergent macroscopic properties of the system were inferred, in statistical terms, from the microscopic properties of the compounding particles. In particular, Boltzmann argued that the macrostate of a system was the average of the different possible molecular microstates, and applied this concept to entropy [11,14,51]. Accordingly, entropy, the most degraded form of energy not able to produce further work was associated with the disorder of the molecules characterizing that specific macrostate, this being the disordered status that was more probable than the ordered one at equilibrium [11,14,51]. Interestingly, Shannon approached information measures following the same idea of the macrostate (information about the source of message) as a function of possible microstates (possible messages sent by the source), consequently deriving a probabilistic definition of entropy (Shannon entropy), closely related to the definition of entropy given by Boltzmann, leading to the observation that the informative content of a system decreases, with the increasing entropy; thus, the disorder of the system [14,15]. Nevertheless, the first to link entropy to information was Leo Szilard, who solved the Maxwell’s demon paradox, suggesting that the molecule sorting activity of such a hypothetic entity (demon) inevitably caused energy expenditure that ultimately contributed to increasing entropy, according to the second law of thermodynamics, which, at that time, Maxwell’s demon paradox seemed to violate [14]. Interestingly, the negative correlations between decreasing Haralick’s “Sum Entropy” (as a measure of the disorder of a vector form the gray level co-occurrence matrix) and increasing fractal dimension (obtained through grayscale differential box counting) was documented in the liver of common carp dosed with perfluorooctanoic acid (PFOA) [20].

Prigogine studied the systems far from (thermodynamic) equilibrium (non equilibrium) and coined the term “dissipative structures”, referring to dynamic open systems displaying self-organization and orderliness; thus, low (internal) entropy. Living organisms, as dynamic complex systems far from equilibrium, behave as dissipative structures, maintaining their internal order (low-entropy) thanks to high-enthalpy, low-entropy intakes from the environment, and returning low-enthalpy, high entropy wastes (heat, excretes, egesta) to the latter [51]. As stressed by Schrödinger, they have to export the entropy they generate to the outer environment in order to live [52]. Interestingly, anatomical structures involved in this “entropy purge” (e.g., lung) show fractal organization [53,54]. Referring to the pathological context, morpho-functional alteration at the different integration scales of living organisms (from subcellular to organismic level) may affect the original complexity and the “entropy purge” needed to maintain the organism far from equilibrium, thus healthy and ultimately alive [20,55]. Death arises as the organism reaches the thermodynamic equilibrium and as its molecules reach the state of maximum (most probable) disorder.

## 3. Pathology and Environmental Pathology

### 3.1. General Pathology

In spite of the multitude of possible causes of diseases (so named noxae), tissue lesions and responses are limited in number and occur according to common pathways. General pathology deals with the latter and may be depicted as the common trunk of the ideal tree, conjoining the roots of basic sciences and biomedical disciplines (physics, chemistry, anatomy, microbiology, physiology, etc.) to the crown of medical clinical disciplines (internal medicine, surgery, etc.), being a multidisciplinary, translational biomedical discipline [56].

From a historical perspective, the masterpiece De sedibus et causis morborum per anatomen indagatis (On the Seats and Causes of Diseases as Investigated by Anatomy) by Giovanni Battista Morgagni represented a milestone in pathology, clearly identifying the pathological basis of diseases at organ level (anatomoclinical correlates) and posing the basis of the modern systemic (special) pathology [57,58,59]. Afterwards, such anatomoclinical correlates were extended down at the tissue level by Xavier Bichat and at cellular level by Rudolph Virchow, who introduced the cellular theory in pathology, identifying the main cell alterations/responses, so founding cellular pathology and the basis of modern medicine [56,58,60]. General pathology (Allgemeine Pathologie) was first introduced in medical courses in Germany in 19th century, searching for the elementary lesions as the basis of diseases. Rudolph Albert Peters introduced the concept of “biochemical lesion” as subcellular metabolic alteration, and Linus Pauling the concept of “molecular pathology”, as an alteration of the configuration of key molecules, altering cell functionality. Summarizing, over the centuries, sick organisms were dissected from the macroscopic down to the molecular level in order to elucidate the pathophysiology associated with each disease, as the pathological alteration of the physiological normal pathways [56]. This frankly reductionistic “molecular dissection” certainly led to important improvement both in terms of diagnosis and therapy; nevertheless, it should be stressed that the object of medicine is primarily to heal individuals, possibly communities and not molecules, because, as taught by complexity science, the individual is greater than the sum of their composing molecules.

### 3.2. Environmental Pathology

Environmental pathology is a branch of general pathology, originally dealing with environmental (climatic, physical) factors acting as a cause of disease. To date, the major interest in environmental pathology is in the study of environmental pollutants as a cause of disease [61,62,63]. As previously stressed, general pathology is a multidisciplinary, translational biomedical discipline; this holds true to a greater extent, if possible, with environmental pathology, due to the close relationships with other related disciplines, such as environmental toxicology, ecotoxicology, and environmental chemistry. Moreover, because environmental pollutants do not act singly and are normally present in the environment at the limit of instrumental detection, a chronic low dose, or possibly a “multiresidual” approach, should be adopted to monitor other environmental and individual parameters (e.g., environmental physico-chemical parameters, age, body parameters, concurrent pathologies, etc.), leading to complex modeling that stresses the need of a complexity-oriented approach [20].

As for other multidisciplinary disciplines, environmental pathology may rely on different techniques, though a morpho-pathological approach represents a good compromise in terms of diagnostic robustness, reliability and affordability, representing, to date, the gold standard in diagnostic pathology. Moreover, in a schematic representation of biomarkers usable for assessing the effects of pollutant stress with increasing ecological relevance and time of response, from the molecular level (timely responsive, but prompt recovering when stressor ceases), up to ecosystem level (slowly responsive and possibly not recovering when stressor ceases), the use of morphological biomarkers from cellular to tissue/organ level may represent a good compromise in terms of response and recovery time, and potential ecological significance [64,65].

#### Image Analysis in Environmental Pathology

Approaching morpho-pathological alterations by means of image analysis can improve discriminative power. The image analysis approach to liver histopathology in common carp exposed to PFOA resulted in a better discrimination among experimental classes compared to high performance liquid chromatography with electro-spray ionization tandem mass spectrometry [66]. Interestingly, though PFOA liver concentrations at environmentally relevant exposure were under the level of detection (LOD), increased expression levels of glutathione S-transferase (GST) gene were documented [67]. Aside from providing information about possible PFOA pathophysiology, the concordance between image analysis and biomolecular data stresses the usefulness of a morpho-pathological, image analysis-based approach in environmental pathology [68].

Referring to other image analysis techniques, advancements in radiological image processing techniques have led to “radiomics” that are able to extract qualitative and quantitative data from clinical images. Thanks to a computational approach, radiomics data may be correlated and integrated with genomic data, resulting in “radiogenomics” as an emerging precision medicine approach [69]. Recently, the opportunity to merge precision medicine data, dealing with singular person health with public health data and to aggregate, integrate and analyze them collectively by means of Big Data tools, has been proposed. Accordingly, the integration of omics, clinical, social, environmental and demographic results in “precision public health bridges the gap between individualized and collective medicine, and gives equal opportunities both to clinicians and public health policy makers [70].

## 4. Perspectives

As previously reported, the evaluation of complexity changes has been assessed, adopting fractal analysis to experimentally document the effect of toxics in aquatic organisms, also at ecologically relevant concentrations [20,21,71]. Moreover, the relationship between complexity changes, in terms of fractal dimension, and informatics entropy has been reported [20]. Intriguingly, the experimental exposure of common carp to PFOA at ecologically relevant concentrations resulted in a counterintuitive increase in complexity, assessed by means of a grayscale differential box counting method, at liver cell level. Such a complexity increase was associated with incipient, mild, reversible cell alteration (cloudy swelling). As a consequence, this histopathological state was regarded as an initial adaptive strategy, a possible hormetic response to cope with toxicant challenge, rather than a mere degenerative, disadaptive status [20,72]. An analogous complexity system increase associated with detoxication and antioxidant protective processes secondary to mild cell stress was observed by Moore as a biphasic or hormetic response in a cell model, adopting network and graph theory. Increasing stress severity and cell injury resulted in cell functional impairment and dysfunction [73]. This resultant correspondence between structural (fractal analysis) and functional (network theory) methods put into perspective the potentialities, either in diagnosis or in research, of the morphological approach to complexity. Traditionally, pathology relies on morphological (histopathological) tissue assessment, though it is rather conservative and reticent to rely on the study of complexity, with the noticeable exception of, among others, Gabriel Landini, who specifically addressed this latter topic in microscopy and image analysis [9,74,75]. Fortunately and currently, digital image analysis is already used in pathology and it will be more widely used in the future [76,77,78]. As a consequence, it would be only a matter of awareness of potentialities related to the study of complexity changes in tissue sections.

Living systems show hierarchic organization levels; therefore, structural and/or functional transition zones are identifiable. Of particular interest are the former; they display clear interface properties and are approachable by means of morpho-pathological, image analysis-based methods. Bilayer membranes are the most pertinent example of such morpho-functional interfaces contributing to subcellular and cellular compartmentalization [79]. Examples of interfaces at the tissue/organ level include lungs, gills, kidney and intestine. Very interestingly, these interfaces display fractal properties and are implied in entropy purge from organisms [53,54], their integrity and functionality being critical for maintaining organisms far from thermodynamic equilibrium [35,52].

**Figure 1:**
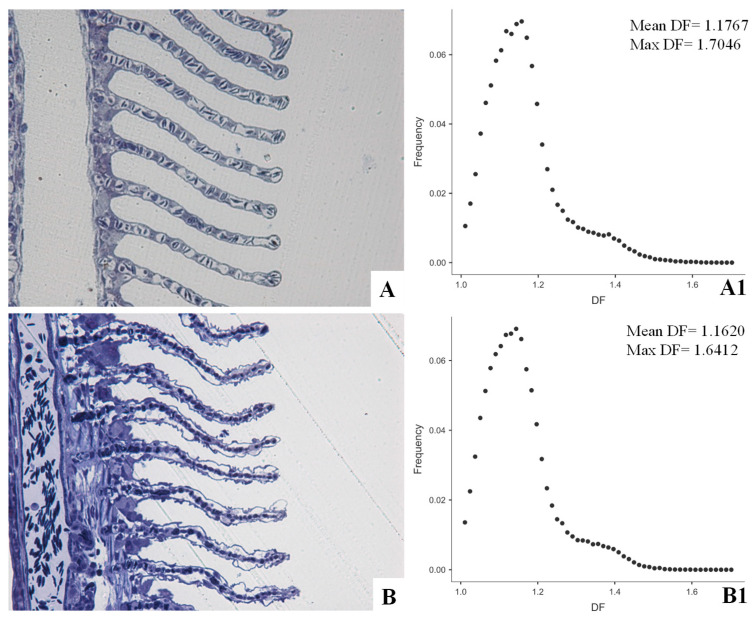
European sea bass, *Dicentrarchus labrax* (Linnaeus, 1758). Epon-Araldite embedded semithin sections stained with Toluidine Blue and observed at light microscopy. Secondary gill lamellae from normal (**A**) and Cd exposed (**B**) exemplars. Cadmium exposure affects both water–epithelial cell interface, where lifting, shrinkage and curling of epithelial cells are appreciable, and pillar cell–blood interface, where pillar cells (gill modified endothelial cells) coarctation results. Morphological evident alterations affect tissue complexity as measured by fractal dimension in binarized, outlined figures. Testing previous tissues for Local Connected Fractal Dimension (LCFD) results in the two scatter plots of frequency (ordinates) to fractal dimension (DF) (abscissa) (**A1**,**B1**). Accordingly, Cd exposure affects mean and maximal fractal dimension. Moreover, a slight left shift of the scatter plot is appreciable compared to normal tissue, resulting in a significant difference (Wilcoxon W, *p* < 0.05) in the paired frequencies. Further information, details about the application of LCFD analysis to gill pathology and about the histopathological, and ultrastructural effects of Cd exposure on European sea bass gills are available in Manera et al. [2,21,81].

From a morphological point of view, biological interfaces are traditionally assessed by means of a qualitative, descriptive histopathological approach. The adoption of morphometric techniques allows approaching them quantitatively (with evident improvement in the statistical evaluation of results) and testing them for possible complexity changes, prone to alter the thermodynamic asset of organism [20,68]. Referring to fish as key indicator organisms for laboratory and field ecotoxicological studies, gills can be referred to as the most extensive interface with aquatic environment and, accordingly, suitable biomarkers for aquatic pollution [80,81]. Though gill assessment is currently used in environmental and ecotoxicological studies, to date there is a lack of standardized methods to objectively quantify gill alterations and, as a consequence, lesions are assessed only qualitatively or semi-quantitatively [82,83]. Nevertheless, metrics, indices, and scores have been proposed to evaluate fish gill status [84,85] and, more recently, the application of the Local Connected Fractal Dimension (LCFD) analysis was proposed as an objective, sensitive and specific method to study gill pathology [21] (Figure 1).

Accounting for local variation in complexity, LCFD is better suited to screen gill than mean global fractal dimension, where opposite local variations may compensate each other on average, masking possible significant differences [21,75,81]. Being an operator-neutral method, it can be implemented and associated with discriminant analysis techniques as possible automated procedures to detect pathological tissue patterns with adequate confidence [21].

In perspective, more attention should be paid to biological interfaces, as complex structures where entropy is transferred among increasing organization levels and purged to the external environment, before they should finally disperse. Where and when this does not happen, a local (intra-organismic) increase in entropy occurs, which leads to complexity reduction, impaired information retention and transmission. Exogenous and/or endogenous toxin retention, and altered energy content use and transfer, will result in an impaired transfer of energy, matter and information, altering all the interfaces down to subcellular molecular level if organism countermeasures (in term of complexity increase) are not adequate to compensate for increased entropy load. This ultimately leads to a frank and chronic pathological state or even death if thermodynamic equilibrium is approached, representing a clear, innovative way to deal with pathology in general and environmental pathology in particular, which is more prone to approaching organisms in a systemic and dynamic view.

## 5. Conclusions

Biomedical disciplines traditionally approach living organisms by means of reductionistic and deterministic methods. In particular, organisms are dissected down to the molecular level in order to gain information about possible molecule to molecule interactions, to derive their macroscopic behavior. Given the complex and chaotic behaviors of living systems, this approach is extremely limited in terms of obtainable information and may lead to misinterpretation.

Approaching environment–organism biological interfaces by means of image analysis tools may result in an effective, objective and operator-neutral way to interpret complexity changes and possible informative content changes at the tissue/organ level and related consequences for the entire organism as an integrated part of the higher hierarchical systems. This approach relies on image analysis methods already in use in digital pathology, though also requires a novel mental paradigm and awareness of the dynamic essence of organisms within ecosystems.

Environmental pathology, as a multidisciplinary discipline, should grant privilege to an integrated, possibly systemic approach that is prone to manage the complex and chaotic aspects which characterize living organisms. Ultimately, environmental pathology should be interested in improving the well-being of individuals, and the population, and ideally the health of the entire ecosystem/biosphere and should not focus merely on single diseases and diseased organs/tissues, cells and/or molecules.

## Data Availability

No new data were created or analyzed in this study. Data sharing is not applicable to this article.
